# Benefit and risk of oral anticoagulant initiation strategies in patients with atrial fibrillation and cancer: a target trial emulation using the SEER-Medicare database

**DOI:** 10.1007/s11239-024-02958-3

**Published:** 2024-03-20

**Authors:** Bang Truong, Lori Hornsby, Brent Fox, Chiahung Chou, Jingyi Zheng, Jingjing Qian

**Affiliations:** 1 Department of Health Outcomes Research and Policy, Auburn University Harrison College of Pharmacy, 4306d Walker Building, Auburn, AL 36849 USA; 2 Department of Pharmacy Practice, Auburn University Harrison College of Pharmacy, Auburn, AL USA; 3 Department of Mathematics and Statistics, Auburn University College of Sciences and Mathematics, Auburn, AL USA

**Keywords:** AFib, Cancer, Oral anticoagulants, Stroke, Bleeding

## Abstract

Oral anticoagulants (OACs) are recommended for patients with atrial fibrillation (AFib) having CHA_2_DS_2_-VASc score ≥ 2. However, the benefits of OAC initiation in patients with AFib and cancer at different levels of CHA_2_DS_2_-VASc is unknown. We included patients with new AFib diagnosis and a record of cancer (breast, prostate, or lung) from the 2012–2019 Surveillance, Epidemiology, and End Results (SEER)-Medicare database (*n* = 39,915). Risks of stroke and bleeding were compared between 5 treatment strategies: (1) initiated OAC when CHA_2_DS_2_-VASc ≥ 1 (*n* = 6008), (2) CHA_2_DS_2_-VASc ≥ 2 (*n* = 8694), (3) CHA_2_DS_2_-VASc ≥ 4 (*n* = 20,286), (4) CHA_2_DS_2_-VASc ≥ 6 (*n* = 30,944), and (5) never initiated OAC (reference group, *n* = 33,907). Confounders were adjusted using inverse probability weighting through cloning-censoring-weighting approach. Weighted pooled logistic regressions were used to estimate treatment effect [hazard ratios (HRs) and 95% confidence interval (95% CIs)]. We found that only patients who initiated OACs at CHA_2_DS_2_-VASc ≥ 6 had lower risk of stroke compared without OAC initiation (HR 0.64, 95% CI 0.54–0.75). All 4 active treatment strategies had reduced risk of bleeding compared to non-initiators, with OAC initiation at CHA_2_DS_2_-VASc ≥ 6 being the most beneficial strategy (HR = 0.49, 95% CI 0.44–0.55). In patients with lung cancer or regional/metastatic cancer, OAC initiation at any CHA_2_DS_2_-VASc level increased risk of stroke and did not reduce risk of bleeding (except for Regimen 4). In conclusion, among cancer patients with new AFib diagnosis, OAC initiation at higher risk of stroke (CHA_2_DS_2_-VASc score ≥ 6) is more beneficial in preventing ischemic stroke and bleeding. Patients with advanced cancer or low life-expectancy may initiate OACs when CHA_2_DS_2_-VASc score ≥ 6.

**Supplementary Information:**

The online version contains supplementary material available at 10.1007/s11239-024-02958-3.

## Introduction

In the United States (US), 2.7–6.1 million people were affected by atrial fibrillation (AFib) annually and it is projected to reach 12 million by 2050 [1]. AFib is associated with more than 454,000 hospitalizations and 158,000 deaths each year [2−4]. Among patients with cancer, AFib was also associated with higher burden of adverse outcomes, such as ischemic stroke, venous thromboembolism (VTE), bleeding, and death compared with AFib patients without cancer [5−8].

Although the benefit of oral anticoagulants (OACs) in patients with AFib has been well established [9], the current management of patients with AFib and cancer regarding OAC treatments remains suboptimal due to insufficient evidence [10]. Among patients with AFib and cancer, OAC initiation was associated with a slightly reduced risk of adverse event (ischemic stroke and intracranial bleeding) compared with non-users [11]. However, recent studies found only half of patients with AFib and cancer initiated OAC, much less than those without cancer [11–14]. One of the major challenges is to determine the appropriate time when patients with AFib and cancer should start OACs to maximize the benefit of stroke prevention while minimizing the risk of bleeding. In general, OAC initiation is recommended for AFib patients with a CHA_2_DS_2_-VASc score ≥ 2, a composite stroke risk score of congestive heart failure, hypertension, age, diabetes mellitus, prior stroke, transient ischemic attack, thromboembolism, vascular disease and sex category [9, 15]. However, such threshold has not been explored in patients with AFib and cancer. For example, when patient with existing cancer is newly diagnosed with AFib with low risk of ischemic stroke (i.e., CHA_2_DS_2_-VASc < 2), whether this patient should start the treatment immediately or wait until they reach a higher risk of ischemic stroke (i.e., CHA_2_DS_2_-VASc ≥ 4 or CHA_2_DS_2_-VASc ≥ 6). In some patient groups, anticoagulation is withheld because of a perceived unfavorable risk-benefit ratio [16]. Since patients with AFib and cancer are at higher risk of stroke and bleeding [5, 6], initiating OAC at low risk may be beneficial in stroke prevention, but may result in increased risk of bleeding. On the other hand, late OAC initiation may prevent risk of bleeding but increase risk of stroke in these patients. Although recent studies found that patients with AFib and cancer who had CHA_2_DS_2_-VASc ≥ 4 were more likely to receive OACs compared to patients with lower risk of stroke [17], the benefit of this treatment strategy has never been explored. Determining the benefit of initiating OACs at different levels of risk of stroke is critically important to optimize the management of patients with AFib and cancer.

In this study, we assessed and compared benefits of multiple OAC initiation treatment strategies at different thresholds of risk of stroke among newly diagnosed AFib patients with cancer using the target trial framework. The target trial framework is the application of design principles from randomized controlled trials (RCTs) to the analysis of observational data to improve the quality of observational epidemiology when a comparator trial is not yet available or feasible [18].

## Materials and methods

### Study design and data source

We used the target trial framework and STrengthening the Reporting of OBservational studies in Epidemiology (STROBE) checklist to conduct and report a retrospective cohort study using the SEER registry linked to the Medicare database (cancer sites: breast, prostate, and lung) from 2011–2019 [19, 20]. The SEER registry contains patient demographics, primary tumor site, tumor characteristics, and cancer stage at diagnosis, treatment, and follow-up of cancer patients across the US [21]. The Medicare data add to SEER data health care services utilization (medical claims, procedures, and prescriptions) [22]. Table 1 summarizes the protocol for target trial and emulation procedure. The study design and study timeline are illustrated by Figure S1.

**Table 1:** Protocol for a target trial and emulation procedure using the SEER-Medicare database

Protocol component	Hypothetical target trial	Emulation in SEER-Medicare data
Eligibility criteria	• Patients aged ≥ 66, newly diagnosed with non-valvular AFib with a history or active breast, lung, or prostate cancer between January 1, 2012 and December 31, 2019. • Beneficiaries continuously enrolled in Medicare part A, B, D, and without Medicare Advantage for 12 months before the diagnosis. • No history of OAC use • No history of mitral valve disease, heart valve repair or replacement, deep vein thrombosis, pulmonary embolism, or joint replacement • Without any diagnosis of stroke within the previous 14 days • Without any conditions associated with an increased risk of bleeding, including: major surgery within the previous month, history of intracranial, intraocular, spinal, retroperitoneal or atraumatic intra-articular bleeding, gastrointestinal hemorrhage within the last 30 days • Without renal impairment stage 5 or end-stage renal diseases within the last 12 months	Same as target trial
Treatment strategies	Eligible individuals are randomly assigned to one of the following 4 treatment strategies (1) initiate OAC when CHA_2_DS_2_-VASc ≥ 1 (2) initiate OAC when CHA_2_DS_2_-VASc ≥ 2 (3) initiate OAC when CHA_2_DS_2_-VASc ≥ 4 (4) initiate OAC when CHA_2_DS_2_-VASc ≥ 6 (5) no initiation of OACs (reference group)	We used cloning-censoring-weighting approach to mimic randomization.
Follow-up	The follow-up of target trial starts at first diagnosis of AFib. End of follow-up is the occurrence of a specific study outcome, the end of administrative censoring (12 months after baseline), death, loss to follow-up, or December 31, 2019, whichever came first.	Same as target trial
Outcomes	Ischemic stroke and major bleeding	Same as target trial
Causal contrast	Intention-to-treat effect, per-protocol effect	Observational analog of per-protocol effect

*AFib* Atrial fibrillation, *OAC* Oral anticoagulants, *CHA*_*2*_*DS*_*2*_*-VASc* A composite score for risk of stroke, *SEER* Surveillance, Epidemiology, and End Results Program

### Study sample and eligibility criteria

#### Study sample

We included individuals aged ≥ 66, newly diagnosed non-valvular atrial fibrillation (NVAF) between January 1, 2012 and December 31, 2019, defined as any International Classification of Disease-9th Revision-Clinical Modification (ICD-9-CM) codes 427.31 or 427.32 or any International Classification of Disease-10th Revision-Clinical Modification (ICD-10-CM) codes I48.xx in any position on one Medicare inpatient claim or on two outpatient claims at least 7 days but < 1 year apart [23]. We retained patients with breast, lung, or prostate cancer—the most common cancer types with AFib—from the SEER file at any time before the initial AFib diagnosis (ICD-O-3 codes C50.0-C50.9 for breast; C34.0, C34.1, C34.2, C34.3, C34.8, C34.9, C33.9 for lung; C61.9 for prostate cancer). Patients were required to continuously enroll in Medicare part A, B, D, and without Medicare Advantage or Health Maintenance Organization (HMO) for at least 12 months before initial NVAF diagnosis.

#### Exclusion criteria

We adapted exclusion criteria from clinical trials [24, 25]. In addition, patients were excluded if they had any other indication than NVAF, contraindication to OACs or had the event of interest shortly before cohort entry: (1) any OAC use during the 12 months baseline period, (2) presence of valvular diseases, repair, or replacement, venous thromboembolism, or joint replacement during the 12 months baseline period, (3) any stroke within 14 days before first NVAF diagnosis, (4) major surgery (i.e., hip fracture, cardiac surgery) or critical bleeding within 30 days before first NVAF diagnosis, (5) renal impairment stage 5 or end-stage renal diseases during the 12 months baseline period. All ICD codes for identification of these conditions can be found in Table S1, Supplementary materials.

### Treatment strategies and assignments

In the hypothetical target trial, eligible individuals were randomly assigned to one of the following 5 treatment strategies: (*Regimen 1*) initiated OAC when CHA_2_DS_2_-VASc ≥ 1, (*Regimen 2*) initiated OAC when CHA_2_DS_2_-VASc ≥ 2, (*Regimen 3*) initiated OAC when CHA_2_DS_2_-VASc ≥ 4, (*Regimen 4*) initiated OAC when CHA_2_DS_2_-VASc ≥ 6, and (*Regimen 5*) never initiated OAC (reference group). In the emulation of target trial, cloning, censoring, and weighting approach were used to mimic the randomization [26]. OAC prescriptions (including warfarin and dabigatran, apixaban, rivaroxaban, edoxaban) were identified from Medicare Part D Prescription Drug Event (PDE) files using national drug code (NDC)) [27]. CHA_2_DS_2_-VASc scores were computed from Medicare claims during12 months before AFib diagnosis and monthly during follow-up, based on a composite of conditions including congestive heart failure (1 point), hypertension (1), age ≥ 75 (2 point), diabetes mellitus (1 point), prior stroke, TIA, or thromboembolism (2 point), vascular disease (e.g. peripheral artery disease, myocardial infarction, aortic plaque) (1 point), age 65–74 years (1 point), and sex category (1 point) [15].

### Follow-up

The follow-up started at the initial NVAF diagnosis (index date) and ended at the occurrence of a study outcome, the end of administrative censoring (12 months after baseline), death (all-cause deaths from the SEER and Medicare files via the variables of “Date of Death Flag”), loss to follow-up (the earliest of 30 days after the end of continuous Medicare Part A, B, or D enrollment or enrollment in an HMO), or December 31, 2019, whichever came first.

### Outcomes

The outcomes of interest were ischemic stroke and major bleeding. We defined major bleeding and ischemic stroke using validated algorithms defined by ICD-9-CM and ICD-10-CM codes in the primary diagnosis from Medicare medical claims files [28, 29, 30].

### Covariates

Covariates selected from prior literature were adjusted in the analysis [24, 28, 31]. Time-fixed baseline covariates were extracted within 12-month period prior to first AFib diagnosis, including: *demographics* (index age, sex, race/ethnicity, calendar year, geographical region, urbanicity), *socioeconomic factors* (household median income, percentage of household with education level below high school, and Medicaid eligibility), *comorbidity risk scores* (CHA_2_DS_2_-VASc, HAS-BLED, and Comorbidity Scores SEER-Medicare version 2021 by NCI) [32], *individual comorbidities* (asthma/chronic obstructive pulmonary disease, hematological disorders, dementia, depression, thrombocytopenia, acute kidney disease (AKD), peptic ulcer disease), *cancer characteristics* (time from cancer diagnosis to the onset of AFib, cancer type, cancer stage, tumor grade, active cancer status [28, 31]), *cancer treatment* (radiation, and cancer-directed surgery, and potentially interacting antineoplastic agents), and *medication history* (angiotensin-converting enzyme inhibitors/angiotensin II receptor blockers, calcium channel blockers, beta blockers, antiarrhythmic medications, diuretics, statin, pump proton inhibitors, and serotonin reuptake inhibitors). Socioeconomic factors such as household income and education level are available at the aggregate area level. If patients had more than one type of cancer before AFib diagnosis, we retained the most recent cancer diagnosis. Cancer treatments were obtained from diagnosis codes or procedures codes within 30 days before AFib diagnosis [33]. Due to high proportion of missing values, other cancer characteristics such as number of regional nodes examined, tumor size, TMN classification, and other cancer-type specific characteristics (i.e., hormone receptor status (HR) and human epidermal growth factor receptor 2 (HER2) for breast cancer or histologic type for lung cancer) were used for descriptive purpose but not adjusted in the models [34]. The following time-varying covariates were extracted monthly after AFib onset, including CHA_2_DS_2_-VASc score, HAS-BLED score, thrombocytopenia, AKD, radiation, cancer-directed surgery, and use of potentially interacting treatment with OACs. These variables may change over time and has an impact on outcomes and the OAC prescription in each month [35, 36]. All diagnosis codes, drug codes, and procedure codes for covariate ascertainment were described in Table S1, Supplementary materials. We used multiple imputation algorithms (fully conditional specification with logistic regression for categorical variables and predictive mean matching for continuous variables) to impute missing values (urbanicity, cancer summary stage, percentage of residents living below poverty, and percentage of non-high school graduates—Table S2, Supplementary Materials) [37].

### Causal contrast

We computed the observational analog of *per-protocol* (PP) effects because cloning-censoring-weighting approach was used [38]. Those who were not compliant to their assigned treatment regimes were censored during follow-up.

### Statistical analysis

Descriptive statistics such as mean and standard deviation (SD) for continuous variables, frequency count and percentage for categorical variables were used to describe the study sample. We quantified the incidence rates of ischemic stroke and major bleeding for each treatment strategy. In the main analysis, cloning-censoring-weighting procedure was used to estimate the treatment effect of 5 treatment strategies [26, 38]. Briefly, we created 5 copies for each individual’s person-time data, then assigned each copy to 5 treatment strategies. *At baseline*, replicates with baseline CHA_2_DS_2_-VASc score that did not comply with their assigned strategy were removed from the dataset. Next, replicates whose data were no longer consistent with their assigned strategy *during follow-up* were censored. To adjust for potential confounding during follow-up, unstabilized time-varying censoring weights were used. Cumulative weights at each time points due to protocol violation are the product of inverse probability of weights for treatment initiation (IPTWs) and inverse probability of censoring weights (IPCWs) due to loss to follow-up (See Technical Appendix). Total weights were truncated at 99th percentile to avoid extreme weights. To estimate the treatment effects for 5 strategies, we fitted a weighted pooled logistic regression estimated by generalized estimating equations (GEEs) with robust variance estimators. We obtained summary hazard ratios (HRs) with 95% confidence interval (95% CIs) and created weighted survival curves comparing four active treatment strategies with the reference strategy. Statistical analyses were conducted using SAS 9.4 (SAS Institute, Inc., Cary, NC, USA).

### Subgroup analyses and sensitivity analyses

We conducted the following subgroup analyses: cancer type (breast, lung, prostate), cancer status at baseline (active, history), cancer stage (in situ, local, regional, and distant), and tumor grade (I, II, and III). In addition, a series of sensitivity analyses were conducted. First, we extended follow-up time to 36 months to explore long-term outcomes for each treatment strategies. Second, since metastatic cancer patients were removed from randomized control trials due to their short live expectancy, we excluded them in this sensitivity analysis [24, 25]. Third, we removed individuals with thrombocytopenia at baseline, since these patients are at elevated risk of bleeding and may not eligible for OAC initiation [39, 40]. Fourth, we further truncated weights at 95th percentile to test the robustness of the treatment effects to the presence of extreme weights [41, 42].

## Results

### Study sample and characteristics

**Fig. 1:**
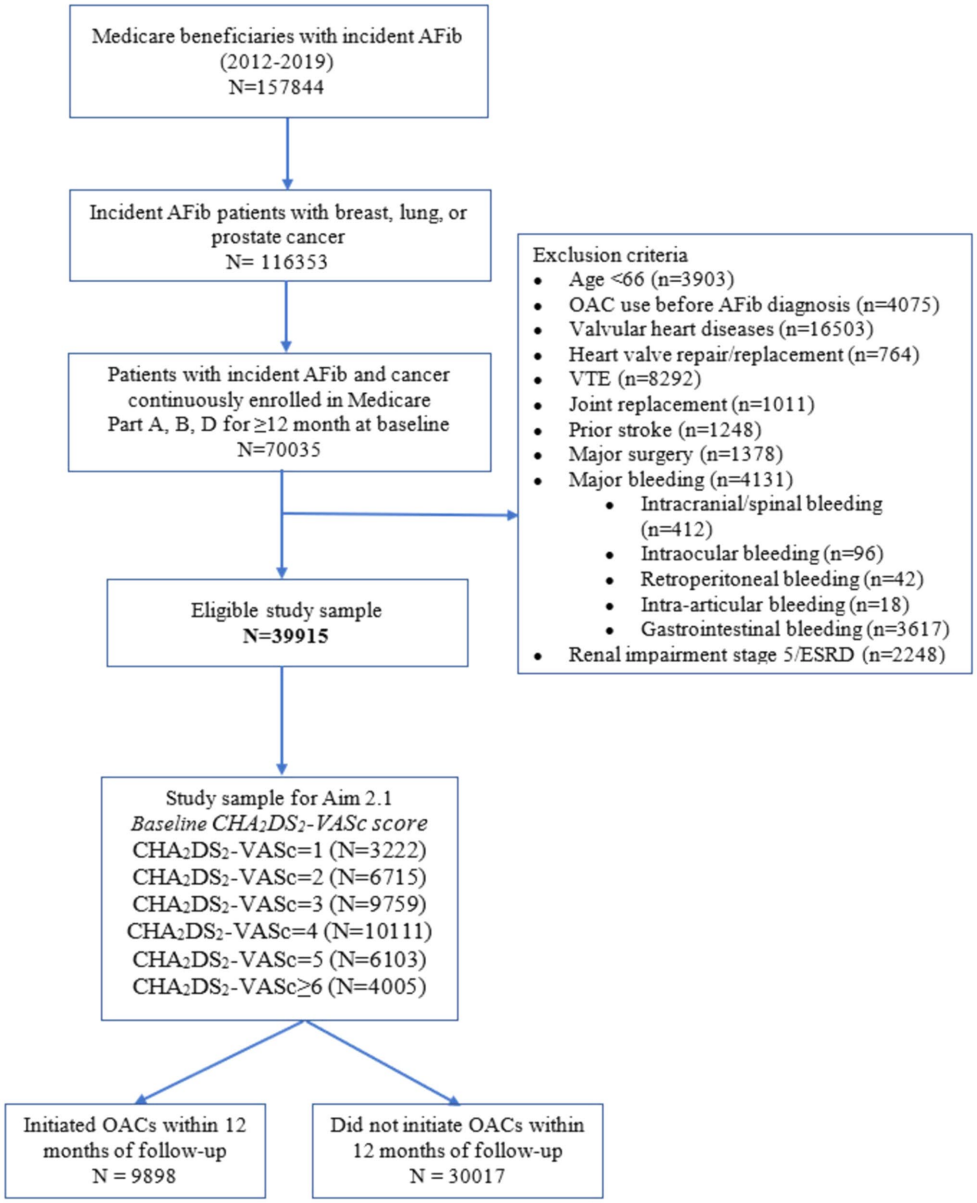
Flowchart diagram for study sample. *VTE* Venous thromboembolism, *AFib* Atrial fibrillation, *OAC* Oral Anticoagulant, *ESRD* End-stage renal diseases

Among 70,035 patients with newly diagnosis of AFib and concomitant cancer in SEER-Medicare data between 2012–2019, the final sample included 39,915 individuals after applying exclusion criteria (Fig. 1). Patients characteristics were described in Table S2. Briefly, study cohort had the mean age of 77.16 (± 7.31) with 46.33% female and the majority were White (85.11%). Regarding cancer characteristics, the majority of the patients had lung cancer (42.85%), with local (47.8%) cancer stage. On average, patients were diagnosed with cancer about 15 months before their AFib onset and 29.06% of them had active cancer. At baseline, the distributions of CHA_2_DS_2_-VASc score at baseline were 3222 patients (8.07%) with the score of 1, 6715 patients (16.82%) with the score of 2, 9759 patients (24.45%) with the score of 3, 10,111 patients (25.33%) with the score of 4, 6103 patients (15.29%) with the score of 5, and 4005 patients (10.03%) with the score of 6 and above. The majority of the cohort has a HAS-BLED of 3 or below (83.76%). Only 9898 patients (24.81%) initiated OACs within 12-month follow-up after their initial NVAF diagnosis (Fig. 1).

### Main analysis

**Fig. 2:**
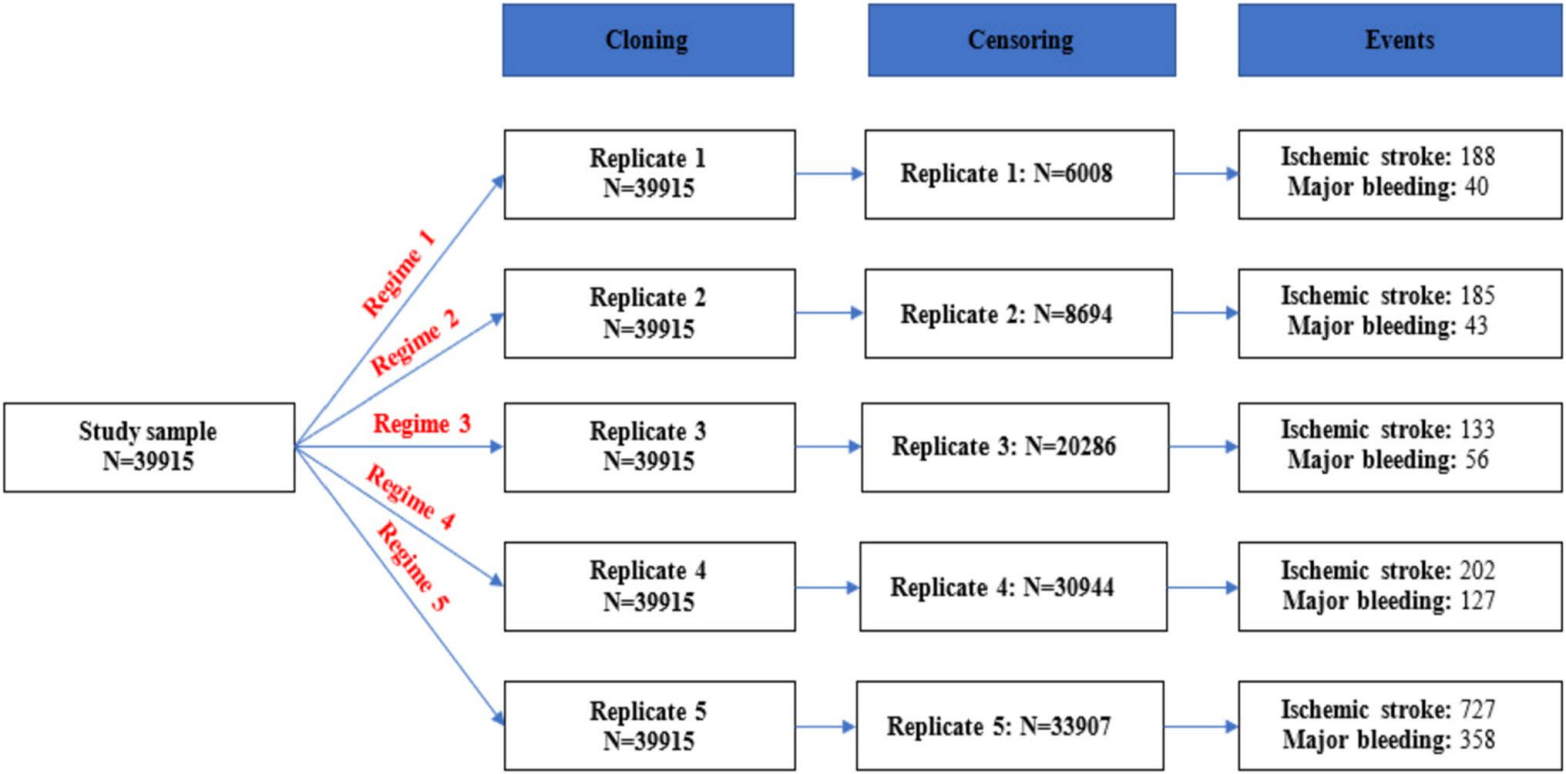
Sample flowchart summarizing cloning and censoring steps

**Fig. 3:**
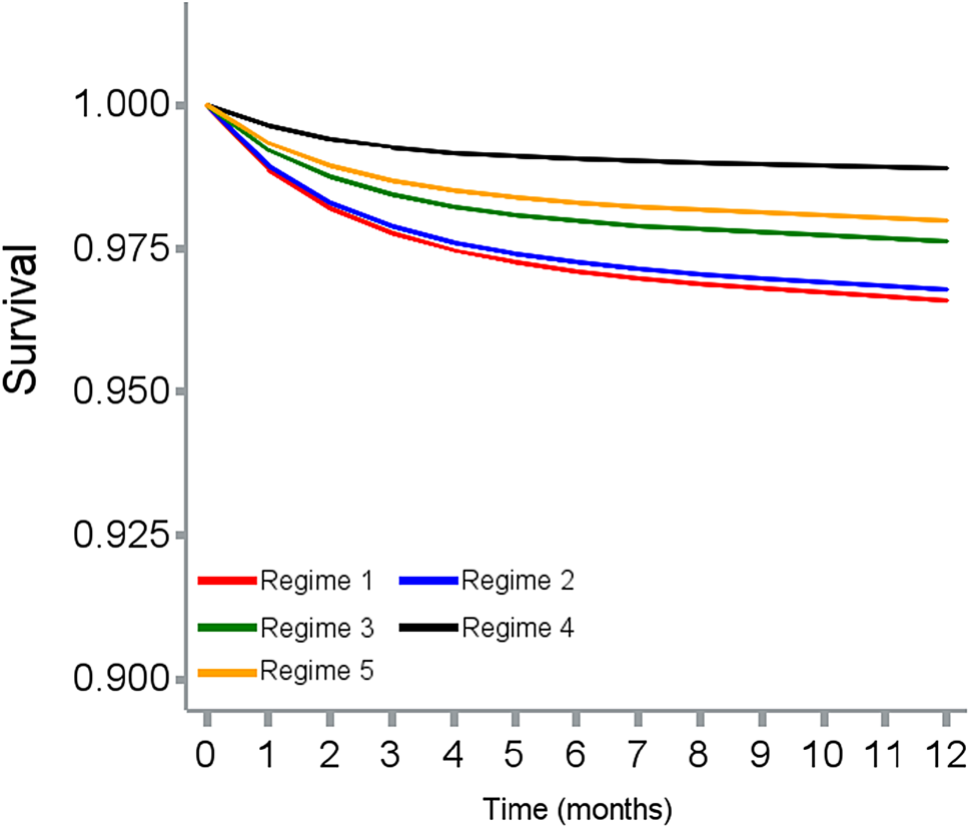
Weighted survival curves for risk of ischemic stroke among 5 treatment regimens. Regimen 1: Initiate oral anticoagulants when CHA_2_DS_2_-VASc score ≥1. Regimen 2: Initiate oral anticoagulants when CHA_2_DS_2_-VASc score ≥2. Regimen 3: Initiate oral anticoagulants when CHA_2_DS_2_-VASc score ≥4. Regimen 4: Initiate oral anticoagulants when CHA_2_DS_2_-VASc score ≥6. Regimen 5: Never initiate oral anticoagulants

**Fig. 4:**
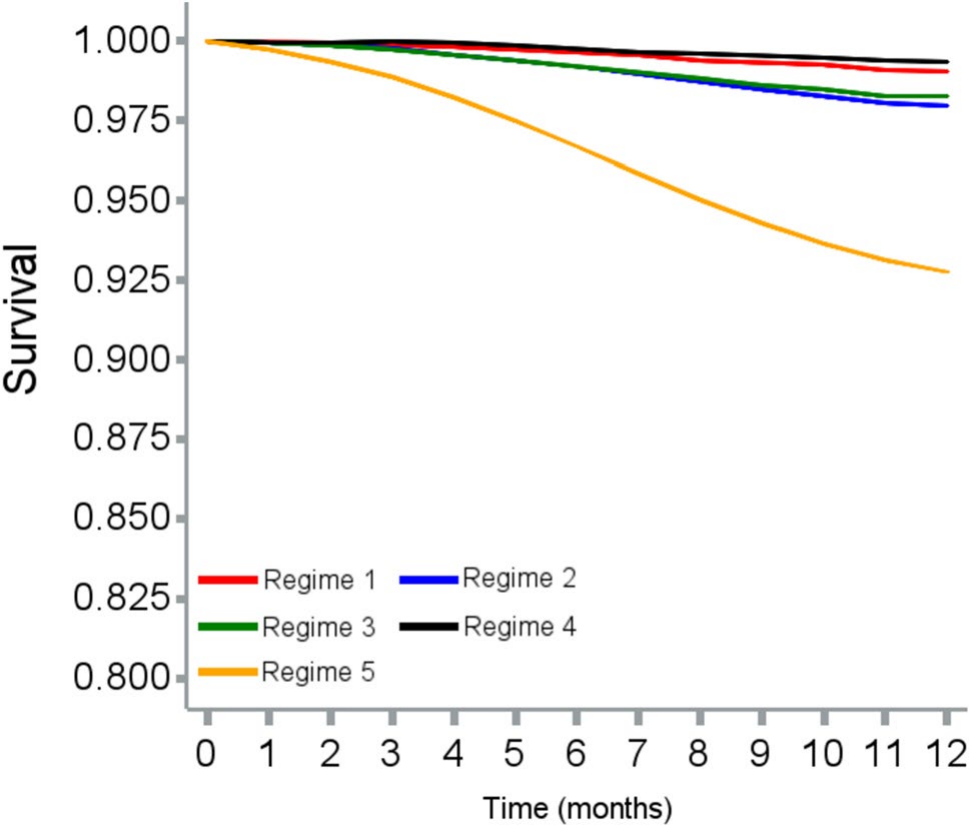
Weighted survival curves for risk of major bleeding among 5 treatment regimens. Regimen 1: Initiate oral anticoagulants when CHA_2_DS_2_-VASc score ≥1. Regimen 2: Initiate oral anticoagulants when CHA_2_DS_2_-VASc score ≥2. Regimen 3: Initiate oral anticoagulants when CHA_2_DS_2_-VASc score ≥4. Regimen 4: Initiate oral anticoagulants when CHA_2_DS_2_-VASc score ≥6. Regimen 5: Never initiate oral anticoagulants

The cloning, censoring, and weighting approach and number of events in each treatment arm are described in Fig. 2. The incidence rates of ischemic stroke and major bleeding (event per person years) for each treatment strategy were 37.75 and 7.87 (*Regimen 1*), 35.03 and 7.90 (*Regimen 2*), 13.88 and 5.81 (*Regimen 3*), 12.18 and 7.64 (*Regimen 4*), and 35.01 and 17.13 (*Regimen 5*). Before weighting, all four OAC initiation strategies (*Regimens 1–4*) reduced risk of bleeding compared with no initiation, while OAC initiation at CHA_2_DS_2_-VASc score ≥ 4 or ≥ 6 (*Regimen 3 and 4*) reduced more ischemic stroke events compared with no initiation. After weighting, only OAC initiation at CHA_2_DS_2_-VASc ≥ 6 (*Regimen 4*) lowered the risk of stroke compared with no initiation (HR = 0.64, 95% CI 0.54–0.75). Other OAC initiation strategies were not beneficial for stroke reduction (*Regimen 1*: HR 1.30, 95% CI 1.11–1.54; *Regimen 2*: HR 1.32, 95% CI 1.12–1.56; *Regimen 3*: HR 1.13, 95% CI 0.95–1.33). All four active treatment regimens reduced the risk of major bleeding, with OAC initiation at CHA_2_DS_2_-VASc ≥ 6 being the most beneficial strategy (HR 0.49, 95% CI 0.44–0.55) (Table 2)**.** The weighted survival curves for each treatment strategy on outcomes of interest are illustrated in Figs. 3 and 4. The distributions of weights are described in Table S3, Figures S2 and S3, Appendix.

**Table 2:** Main analysis comparing 5 treatment regimens of oral anticoagulation initiation in patients with atrial fibrillation and cancer

	Regimen 1	Regimen 2	Regimen 3	Regimen 4	Regimen 5
Ischemic stroke					
Event	188	185	133	202	727
Person-years	4980	5282	9480	16,589	200,767
Incidence rate per 1000 person-year	37.75	35.03	13.88	12.18	35.01
Unadjusted HR (95% CI)	1.15 (0.99–1.34)	1.02 (0.88–1.18)	**0.45 (0.39–0.51)**	**0.41 (0.38–0.45)**	Reference
Adjusted HR (95% CI)	**1.30 (1.11–1.54)**	**1.32 (1.12–1.56)**	1.13 (0.95–1.33)	**0.64 (0.54–0.75)**	Reference
Major bleeding					
Event	40	43	56	127	358
Person-years	5084	5382	9644	16,629	20,898
Incidence rate per 1000 person-year	7.87	7.90	5.81	7.64	17.13
Unadjusted HR (95% CI)	**0.45 (0.35–0.60)**	**0.45 (0.34–0.58)**	**0.39 (0.33–0.46)**	**0.53 (0.48–0.58)**	Reference
Adjusted HR (95% CI)	**0.55 (0.38–0.78)**	**0.61 (0.42–0.87)**	**0.58 (0.41–0.81)**	**0.49 (0.44–0.55)**	Reference

Bold values indicate statistically significant treatment effects

The final models incorporated inverse probability of treatment weights (IPTWs, adjusted for baseline, time-varying covariates, and treatment history) and inverse probability of censoring weights (IPCWs, adjusted for baseline, time-varying covariates, and treatment history)

Regimen 1: Initiate oral anticoagulants when CHA_2_DS_2_-VASc score ≥ 1

Regimen 2: Initiate oral anticoagulants when CHA_2_DS_2_-VASc score ≥ 2

Regimen 3: Initiate oral anticoagulants when CHA_2_DS_2_-VASc score ≥ 4

Regimen 4: Initiate oral anticoagulants when CHA_2_DS_2_-VASc score ≥ 6

Regimen 5: Never initiate oral anticoagulants

*HR* Hazard ratio, *CI* Confidence Interval

### Subgroup and sensitivity analyses

The main findings were consistent across subgroups of patients with active/inactive cancer status. In addition, starting at CHA_2_DS_2_-VASc ≥ 6 remained the most beneficial regimens across all subgroups. However, there were some heterogeneity of treatment effects in other subgroups. Specifically, in patients with short life expectancy or advanced cancer such as lung cancer and regional/metastatic cancer, OAC initiation at any CHA_2_DS_2_-VASc level increased risk of stroke and did not reduce risk of bleeding (except for starting at CHA_2_DS_2_-VASc ≥ 6). For instance, HRs of OAC initiation at CHA_2_DS_2_-VASc ≥ 1 (Regimen 1) were 2.10 (95% CI 1.55–2.86) for ischemic stroke and 0.83 (95% CI 0.43–1.64) for major bleeding among lung cancer patients. In metastatic cancer patients, the corresponding HRs were 2.09 (95% CI 1.33–3.28) and 1.17 (95% CI 0.48–2.90). In other subgroups (i.e., breast cancer, prostate cancer, in situ/local stage, grade I/II/III), OAC initiation at any level did not increase risk of stroke while reduced risk of bleeding compared with no initiation (Table S4, Appendix). In sensitivity analyses, the main findings remained robust when extending follow-up to 36 months, removing high-risk patients, and in the absence of extreme weights (Table S5, Appendix).

## Discussion

Our study is the among the first to assess the benefit of OAC initiation in patients with AFib and cancer at different level of risk for stroke. First, we found that initiating OACs at higher level of CHA_2_DS_2_-VASc score (i.e., ≥ 6) is more beneficial in reducing risk of stroke among patients with AFib and cancer. OAC initiation at a lower level of CHA_2_DS_2_-VASc score might be harmful or has no effect on risk of stroke. Second, initiating OACs at any level of CHA_2_DS_2_-VASc score reduced the risk of major bleeding, with OAC initiation at higher level of CHA_2_DS_2_-VASc score being the most effective strategy. Thus, among cancer patients with new AFib diagnosis, OAC initiation may be considered for patients at high risk of stroke (CHA_2_DS_2_-VASc score at least ≥ 4) when a marginal harm on risk of stroke and a benefit on risk on bleeding are observed. In addition, among patients with advanced cancer status or low life-expectancy (i.e., lung cancer or regional/metastatic cancer), OAC should be given only to patients with CHA_2_DS_2_-VASc score ≥ 6.

OACs were underutilized in the management of patients with AFib and cancer in previous studies [12, 17]. In fact, we found that only one in four patients initiated OACs within the first year after AFib diagnosis in this study. While current guidelines recommend a CHA_2_DS_2_-VASc score ≥ 2 for OAC initiation in general AFib patients [9], this threshold may not be applicable for patients with cancer because they are at higher risk of stroke and bleeding [5–8]. In this study, we found that OAC initiation at higher CHA_2_DS_2_-VASc score (6 or above) was the most beneficial treatment strategy. Starting OACs at lower CHA_2_DS_2_-VASc score may not be beneficial for stroke reduction within one year after AFib diagnosis. The treatment effects sustained after 3 years of follow-up in the sensitivity analysis. In addition, starting OAC at any CHA_2_DS_2_-VASc level was associated with reduced risk of bleeding compared with no initiation.

Similar to our findings, Atterman (2020) found that OAC initiation was associated with a slightly reduced risk of ischemic stroke and intracranial bleeding compared with non-users (HR = 0.90 95% CI 0.80-1.00), especially in those with moderate (HR 0.82, 95% CI 0.70–0.96) or high (HR 0.82, 95% CI 0.79–0.86) baseline CHA_2_DS_2_-VASc score [11]. The authors obtained CHA_2_DS_2_-VASc score before AFib diagnosis (index date) and stratified the risk of stroke based on baseline CHA_2_DS_2_-VASc score (0: low, 1: intermediate, ≥ 2: high) [11]. Likewise, O’Neal (2018) found AFib patients with cancer who sought for cardiologists shortly after AFib diagnosis were more likely to receive OACs and had a reduced risk of stroke and non-inferior risk of bleeding compared with those who did not [14]. Recent studies also advocated the use of OACs in patients with AFib and cancer having CHA_2_DS_2_-VASc 0-2 [43, 44]. Leader (2023) showed that 12-month cumulative incidence of arterial thromboembolism was higher in patients with the AFib and cancer compared to patients with AFib and no cancer not receiving OACs [43]. Indeed, these studies compared the incidence of stroke and bleeding between OAC users and non-users and stratified the comparison by baseline CHA_2_DS_2_-VASc score or compared the risks of stroke or bleeding between patients with AFib and cancer versus patients with AFib and no cancer, but did not directly compare risk of outcomes between different OAC initiation strategies based on their CHA_2_DS_2_-VASc score as time-varying confounder during follow-up [11, 14, 43]. In addition, such design is subjected to immortal time bias since patients would have been stroke-free or bleeding-free long enough to receive OACs [45]. Our study clearly formulated a decision point where cancer patients newly diagnosed with AFib should start treatment at lower risk of stroke or wait until they reach a higher risk level. We used cloning-censoring-weighting approach to assign each patient into different treatment strategies and followed patients after their AFib diagnosis, which minimized immortal time bias by accounting for patient’s exposure to OACs and the compliance with their assigned treatment during follow-up [26, 46].

We found that the effects of OAC initiation at different risk level based on CHA_2_DS_2_-VASc score were heterogeneous in several subgroups of cancer. In patients with advanced cancer such as lung cancer or regional/metastatic cancer, OAC initiation may not be beneficial or even harmful in patients with a lower risk of stroke, but beneficial only in patients with high risk of stroke. In subgroups of cancer such as breast cancer or prostate cancer, in situ/local cancer or grade I/II/III, OAC initiation at lower CHA_2_DS_2_-VASc score did not increase risk of stroke but decreased risk of bleeding, while OAC initiation at higher CHA_2_DS_2_-VASc score decreased risk of stroke and bleeding compared with no initiation. This heterogeneity can be explained by the differential risk of stroke and bleeding in patients at different stages of cancer. Indeed, patients with advanced cancer (i.e., metastatic cancer or lung cancer) are at higher risk of stroke and bleeding compared with early stage [47, 48]. In the sensitivity analyses, starting OAC at any CHA_2_DS_2_-VASc score was associated with a non-inferior risk of stroke and lower risk of bleeding after excluding patients with metastatic cancer at baseline. These findings may help clinicians tailorize OAC treatment strategy in AFib patients based on their cancer characteristics.

CHA_2_DS_2_-VASc score has been used for more than a decade for risk of stroke stratification and OAC initiation in patients diagnosed with AFib. In this study, we used CHA_2_DS_2_-VASc as an indicator for OAC initiation although the tool was found not highly predictive in stroke prediction in patients with AFib and cancer [49, 50]. CHA_2_DS_2_-VASc score has shown low discrimination capacity for ischemic stroke in patients with AFib and cancer than patients without cancer [50, 51]. The major limitation of using CHA_2_DS_2_-VASc score is that it is not able to capture an independent risk of stroke caused by cancer, especially in patients with advanced cancer [49, 52]. In fact, CHA_2_DS_2_-VASc thresholds for each treatment strategy in our study were selected based on the distribution of baseline CHA_2_DS_2_-VASc scores of the study sample and in prior study [17]. Indeed, all patients enrolled in this study had CHA_2_DS_2_-VASc ≥ 1. In general AFib patients, OACs are recommended for those with CHA_2_DS_2_-VASc ≥ 2 [9, 53, 54]. In addition, recent studies have shown patients with CHA_2_DS_2_-VASc ≥ 4 or ≥ 6 were more likely to initiate OACs [17]. Therefore, our choice of CHA_2_DS_2_-VASc thresholds for each treatment strategy reflects multiple scenarios for OAC initiation in patients with AFib and cancer: prescribe OACs for all patients regardless their risk of stroke; prescribe OACs based on general AFib recommendations; and real-world pattern of OAC use in clinical practice. However, CHA_2_DS_2_-VASc score has been widely accepted among clinicians and recommended in clinical guidelines for OAC initiation decision-making [9, 54, 55]. There is an urgent need to develop new tools for risk of stroke assessment in patients with AFib and cancer.

Our study is subject to some limitations. First, unmeasured confounding such as patients’ frailty, body mass index could not be captured by SEER-Medicare data. In addition, cancer characteristics used in the analysis such as cancer stage and tumor grade were captured at the time of cancer diagnosis rather than at the time of AFib diagnosis since the SEER registry is lack of measurements of progression of cancer characteristics (cancer stage, tumor grade) over time. We also could not control for some cancer-specific characteristics such as receptor status (ER, HER2) for breast cancer, histological type, or tumor size in our analysis since they contained large proportions of missing values. In addition, we assumed 12-month baseline period prior to AFib diagnosis was sufficient to capture patients’ baseline characteristics, therefore, measurement bias may persist. Measurement bias was also present when we measured patients’ behavioral risk factors (i.e., alcohol use disorders in HAS-BLED score) using ICD codes [56]. Also, socioeconomic factors from Census tract were not available on an individual level. Third, residual bias could not be completely eliminated even though we used validated algorithms to define eligibility criteria and outcomes. Fourth, we did not stratify OAC initiation by type of OACs (i.e., warfarin, dabigatran, rivaroxaban) given their safety and effectiveness profile may be different. An updated meta-analysis of RCTs showed better efficacy and safety of DOACs than warfarin [57]. while a recent observational study using SEER-Medicare data found warfarin and DOACs are equivalently safe and effective in prevention stroke and bleeding [58]. Thus, the stratified treatment effects may be different than the marginal effects of all OACs and the benefits and risks of different OAC initiation strategies in this study may be biased depending on which type of OACs were used. Fourth, since we cloned each individuals to 5 copies, the 95% CI estimated from GEEs might be conservative due to correlation between clones. In our analysis, we could not perform non-parametric bootstrapping to obtain 95% CIs due to computational time. Fifth, our estimates may be prone to bias in the presence of extreme weights although we truncated the initial weights at 99th percentile [59]. Using stabilized variance may reduce the variance and avoid extreme weights, but the stabilization procedures might not valid for cloning-censoring-weighting approach like our study [60]. However, the results remain robust in the sensitivity analysis after we truncated the weights to 95th percentile. Sixth, our findings may not be generalizable beyond the target population in this study (i.e., patients with newly diagnosed cancer on existing AFib, other cancer types, or non-Medicare populations). Future studies are warranted to investigate the benefits and risks of OACs among patients with other advanced cancer such as hematological cancers due to higher risk of stroke and bleeding in this population [61, 62].

Although our study emulated a hypothetical target trial and adopted components (i.e., inclusion/exclusion criteria, outcomes, and follow-up) from prior RCTs [24, 25], several components were not perfectly mimicked. Specifically, RCTs removed patients platelet count < 90,000/µL, systolic blood pressure ≥ 180 mmHg or diastolic blood pressure ≥ 100 mmHg, or creatinine clearance less than 30 mL/min at the screening visit [24, 25]. However, these lab values were not available in SEER-Medicare data. We therefore replaced these conditions with the presence of thrombocytopenia or severe renal impairments. In addition, several components were defined by clinicians’ assessment in RCTs, such as AFib definition by an electrocardiogram (ECG) document or congestive heart left failure with ventricular ejection fraction ≤ 35% [24, 25]. Moreover, therapeutic responses and adverse events were monitored with international normalized ratio (INR) and liver-function tests, which are not available in our emulation [24, 25]. Although non-randomization component has been criticized as the main source of bias in observational studies, it was not proven as the primary cause of inconsistency between observational and RCTs. Successful emulation without randomization has been conducted to benchmark the estimates from observational studies to RCTs and vice versa, especially during the COVID pandemic when the need of RCTs could not be met due to time constraint [63–65]. In this study, randomization was assumed using a cloning-censoring-weighting approach and the adjustment of measured time-varying confounding during follow-up [26]. It is also necessary to highlight that misspecification of time zero has been found as the major source of failure in obtaining valid causal effects in observational studies [19, 20]. In our study, we specified time zero by aligning the time when all inclusion and exclusion criteria met, start of treatment strategies, and follow-up. Such practice removed immortal time bias and prevalent user bias from our analysis [19, 20].

Our study has many strengths. Using the target trial emulation framework to design the study and the cloning–censoring–weighting approach, we explicitly designed a trial to answer a causal question. We included patients with newly AFib diagnosis and followed them after AFib diagnosis to remove survival bias. In addition, we further adjusted for important confounders such as cancer characteristics by the linkage between Medicare administrative claims data and the SEER registry. We pre-specified a wide range of subgroup analyses and sensitivity analyses to confirm the robustness of the main analysis. Our findings are expected to help clinicians’ decision making in optimizing OAC initiation and individualizing their decisions based on patient’s cancer characteristics.

## Conclusion

Among cancer patients with new AFib diagnosis, OAC initiation at higher risk of stroke (CHA_2_DS_2_-VASc score ≥ 6) may be more beneficial in preventing ischemic stroke and bleeding. Patients with advanced cancer status or low life-expectancy may initiate OACs when CHA_2_DS_2_-VASc score ≥ 6.

### Supplementary Information

Below is the link to the electronic supplementary material.

Supplementary material 1 (DOCX 1576.3 kb)

Supplementary material 2 (DOC 91.0 kb)
