# Supplementary material for: Benefit and risk of oral anticoagulant initiation strategies in patients with atrial fibrillation and cancer: a target trial emulation using the SEER-Medicare database
Source: J Thromb Thrombolysis. 2024 Mar 20;57(4):638–49. doi: 10.1007/s11239-024-02958-3 (PMC11026243; doi:10.1007/s11239-024-02958-3)
Supplement: Supplementary file 1 — Supplementary material 1 (DOCX 1576.3 kb) [file 11239_2024_2958_MOESM1_ESM.docx]

**Benefit and risk of oral anticoagulant initiation strategies in patients with atrial fibrillation and cancer: a target trial emulation using the SEER-Medicare database**

Bang Truong, MS; Lori Hornsby, PharmD^,^ Brent Fox, PharmD, PhD; Chiahung Chou, PhD; Jingyi Zheng, PhD; Jingjing Qian, PhD.

**Supplementary materials (tables, figures)**

**Table S1.** Algorithms to identify study components from SEER-Medicare data

| **Component** | **Code type** | **Codes** |
| --- | --- | --- |
| ***Eligibility criteria*** | | |
| AFib | ICD-9-CM | 427.31 or 427.32 |
|  | ICD-10-CM | I48.xx |
| Breast cancer | ICD-O-3 | C50.0-C50.9 |
| Lung cancer | ICD-O-3 | C34.0, C34.1, C34.2, C34.3, C34.8, C34.9, C33.9 |
| Prostate cancer | ICD-O-3 | C61.9 |
| Valvular heart diseases | ICD-9-CM | 0932, 394, 395, 396, 3970, 3971, 240, 4241, 242, 4243, 7460, 7461, 7462, 7463, 7464, 7465, 7466, 99602, 99671, V422 |
|  | ICD-10-CM | I05, I06, I07, I08, I34, I35, I36, I37, I39, Q22, Q23, T820, T8201, T8202, T8203, T8209, T8222, T826, Z952, Z953, Z954 |
|  | ICD-9-CM PX | 351, 352, 3533, 3595, 3599 |
|  | ICD-10-PCS | 02RF, 02RG, 02RH, 02RJ, 02QF, 02QG, 02QH, 02QJ |
| Heart valve repair or replacement | ICD-9-CM | V433 |
| Venous thromboembolism | ICD-9-CM | 4151 ,453, V1251, V1255 |
|  | ICD-10-CM | I26, I80, I81, I82, Z8671 |
| Joint replacement | ICD-9-CM PX | 8151, 8152, 8154 |
|  | ICD-10-PCS | 0SR9019, 0SR901A, 0SR901Z, 0SR9029, 0SR902A, 0SR902Z, 0SR9039, 0SR903A, 0SR903Z, 0SR9049, 0SR904A, 0SR904Z, 0SR9069, 0SR906A, 0SR906Z, 0SR907Z, 0SR90EZ, 0SR90J9, 0SR90JA, 0SR90JZ, 0SR90KZ, 0SRA009, 0SRA00A, 0SRA00Z, 0SRA019, 0SRA01A, 0SRA01Z, 0SRA039, 0SRA03A, 0SRA03Z, 0SRA07Z, 0SRA0J9, 0SRA0JA, 0SRA0JZ, 0SRA0KZ, 0SRB019, 0SRB01A, 0SRB01Z, 0SRB029, 0SRB02A, 0SRB02Z, 0SRB039, 0SRB03A, 0SRB03Z, 0SRB049, 0SRB04A, 0SRB04Z, 0SRB069, 0SRB06A, 0SRB06Z, 0SRB07Z, 0SRB0EZ, 0SRB0J9, 0SRB0JZ, 0SRB0KZ, 0SRE009, 0SRE00A, 0SRE00Z, 0SRE019, 0SRE01A, 0SRE039, 0SRE03A, 0SRE03Z, 0SRE07Z, 0SRE0J9, 0SRE0JA, 0SRE0JZ, 0SRR019, 0SRR01A, 0SRR01Z, 0SRR039, 0SRR03A, 0SRR03Z, 0SRB0JA, 0SRR07Z, 0SRR0J9, 0SRR0JA, 0SRR0JZ, 0SRR0KZ, 0SRS019, 0SRE01Z, 0SRS01A, 0SRS01Z, 0SRS039, 0SRS03A, 0SRS03Z, 0SRS07Z, 0SRE0KZ,  0SRS0J9, 0SRS0JA, 0SRS0JZ, 0SRS0KZ, 0SRC069, 0SRC06A, 0SRC06Z, 0SRC07Z, 0SRC0EZ, 0SRC0J9, 0SRC0JA, 0SRC0JZ, 0SRC0KZ, 0SRC0L9, 0SRC0LA, 0SRC0LZ, 0SRC0M9, 0SRC0MA, 0SRC0MZ, 0SRC0N9, 0SRC0NA, 0SRC0NZ, 0SRD069, 0SRD06A, 0SRD06Z, 0SRD07Z, 0SRD0EZ, 0SRD0J9, 0SRD0JA, 0SRD0JZ, 0SRD0KZ, 0SRD0L9, 0SRD0LA, 0SRD0LZ, 0SRD0M9, 0SRD0MA, 0SRD0MZ, 0SRD0N9, 0SRD0NA, 0SRD0NZ, 0SRT07Z, 0SRT0J9, 0SRT0JA, 0SRT0JZ, 0SRT0KZ, 0SRU07Z, 0SRU0J9, 0SRU0JA, 0SRU0JZ, 0SRU0KZ, 0SRV07Z, 0SRV0J9, 0SRV0JA, 0SRV0JZ, 0SRV0KZ, 0SRW07Z, 0SRW0J9, 0SRW0JA, 0SRW0JZ, 0SRW0KZ |
| Renal impairment stage 5/ESRD | ICD-9-CM | 40301, 40311, 40391, 5855, 5856, V451, V56 |
|  | ICD-10-CM | I120, I1311, I132, N185, Y841, Z49, Z9115, Z992 |
|  | ICD-9-CM PX | 3995, 5498 |
|  | ICD-10-PCS | 3E1M39Z, 5A1D70Z, 5A1D80Z, 5A1D90Z |
| History of stroke/TIA | ICD-9-CM | 36231, 36232, 36233, 36234, 43301, 43311, 43321, 43331, 43381, 43391, 43401, 43411, 436, 430, 431, 43391, 435 |
|  | ICD-10-CM | H340, H341, H342, I63, I60, I61, I62, I63, G45, I6782, I6789 |
| Major surgery |  | Not included due to large number of codes |
| Intracranial/Spinal bleeding | ICD-9-CM | 430, 431, 432, 852, 853, |
|  | ICD-10-CM | I60, I61, I62, S064, S065, S066 |
| Intraocular bleeding | ICD-9-CM | 36281, 37923, 36361, 36362, |
|  | ICD-10-CM | H356, H3130, H3131, H431 |
| Retroperitoneal bleeding | ICD-9-CM | 56881 |
|  | ICD-10-CM | K661 |
| Atraumatic intra-articular bleeding | ICD-9-CM | 7191 |
|  | ICD-10-CM | M250 |
| Gastrointestinal bleeding | ICD-9-CM | 4560, 45620, 5301, 5307, 53082, 5310, 5311, 5312, 5313, 5314, 5315, 5316, 5317, 5319, 5320, 5321, 5322, 5323, 5324, 5325, 5326, 5327, 5329, 5330, 5331, 5332, 5333, 5334, 5335, 5336, 5337, 5339, 5340, 53400, 53401, 5341, 5342, 5343, 5344, 5345, 5346, 5347, 5349, 53500, 53501, 53510, 53511, 53520, 53521, 53530, 53531, 53540, 53541, 53550, 53551, 53560, 53561, 53783, 5780, 4551, 4552, 4554, 4555, 4556, 4557, 4558, 4559, 56200, 56201, 56202, 56203, 56210, 56211, 56212, 56213, 5693, 56985, 5781, 5789 |
|  | ICD-10-CM | I8501, I8511, K20, K210, K2211, K226, K250, K251, K252, K254, K255, K256, K260, K261, K262, K264, K265, K266, K270, K271, K272, K274, K275, K276, K280, K281, K282, K284, K285, K286, K2901, K2921, K2931, K2941, K2951, K2961, K2971, K2981, K2991, K31811, K920, K5521, K5701, K5711, K5721, K5731, K5741, K5751, K5753, K5781, K5791, K5793, K625, K640, K641, K642, K643, K644, K645, K648, K649, K921, K922 |
| ***Outcomes*** | | |
| Ischemic stroke (new diagnosis) | ICD-9-CM | 36231, 36232, 36233, 36234, 43301, 43311, 43321, 43331, 43381, 43391, 43401, 43411, 436, H340, H341, H342, I63 |
|  | ICD-10-CM |  |
| Major bleeding | ICD-9-CM | 3361, 36361, 36362, 36372, 37632, 37742, 37923, 4230, 430, 431, 432, 56881, 7191, 72992, 852, 853, 86601, 86602, 86611, 86612 |
|  | ICD-10-CM | G9519, H0523, H3130, H3131, H3141, H431, H4702, I230, I312, I60, I61, I62, K661, M250, M7981, S064, S065, S066, S260, S3701, S3702, S3703, S3704, S3705, S3706 |
| ***Covariates*** | | |
| CHF | ICD-9-CM | 39891, 40201, 40211, 40291, 40401, 40403, 40411, 40413, 40491, 40493, 4254, 4259, 428 |
|  | ICD-10-CM | I0981, I110, I130, I132, I425, I428, I50 |
| HTN | ICD-9-CM | 401, 402, 403, 404, 405 |
|  | ICD-10-CM | I10, I11, I12, I13, I14, I15, I16 |
| DM | ICD-9-CM | 250, 3572, 3620, 36641, |
|  | ICD-10-CM | E10, E11, E13 |
| Vascular diseases | ICD-9-CM | 410, 412, 4400, 4402, 4403, 4409, 4442, 4439, 44481 |
|  | ICD-10-CM | I21, I252, I700, I702, I703, I704, I705, I706, I707, I709, I742, I743, I744, I739, I745 |
| Renal diseases | ICD-9-CM | 0160, 0954, 1890, 1899, 2230, 23691, 2504, 2714, 2741, 28311, 403, 404, 4401, 4421, 4473,  5724, 580, 581, 582, 583, 584, 585, 586, 587, 588, 591, 6421, 6462, 75312, 75313, 75314, 75315, 75316, 75317, 75319, 7532, 7944, V420, V451, V56 |
|  | ICD-10-CM | A1811, A5275, C649, C689, D4100, E1129, E1029, E1121, E1021, E748, M1030, N200, D593, I120, I129, I1310, I130, I1311, I132, I1311, I701, I722, I773, K767, N00, N01, N02, N03, N04, N05, N06, N07, N08, N1330, O10419, O10411, O10412, O10413, O1042, O1043, O26839, O1214, O26831, O26832, O26833, Q613, Q612, Q6119, Q614, Q615,  Q6102, Q618, Q6239, Q6211, Q6212, Q6231, Q6210, Q6211, R944, Z940, Z992, Z9115, Z4931, Z4901, Z4902, Z4932 |
| Liver diseases | ICD-9-CM | 070, 07271, 09162, 1305, 571, 573, 7948 |
|  | ICD-10-CM | A5145, B0081, B15, B16, B17, B18, B19, B251, B2681, B581, B942, K70, K71, K72, K73, K74, K75, K76, K77, R94.5 |
| Bleeding disposition | ICD-9-CM | 430, 431, 432, 56881, 5997, 5307, 5310, 5312, 5314, 5316, 5320, 5322, 5324, 5326, 5330, 5332, 5334, 5336, 5340, 5342, 5344, 5346, 5693, 53501, 53511, 53521, 53531, 53541, 53551, 53561, 53571, 53783, 53784, 56202, 56203, 56212, 56213, 56985, 578, 7847, 7863, 6262, 7191, 37272, 459 |
|  | ICD-10-CM | I60, I61, I62, K661, R31, K226, K250, K252, K254, K256, K260, K262, K264, K266, K270, K272, K274, K276, K280, K282, K284, K286, K625, K2901, K2921, K2931, K2941, K2951, K2961, K2971, K2981, K2991, K31811, K3182, K5701, K5711, K5713, K5721, K5731, K5733, K5741, K5751, K5753, K5781, K5791, K5793, K5521, K920, K921, K922, R040, R042, N920, M250, M122, H113, R58 |
| Alcohol use disorders | ICD-9-CM | 291, 303, 3050, 3575, 4255, 5353, 5710, 5711, 5712, 5713, 7903 |
|  | ICD-10-CM | E8600, V113, F10, Z714 |
| Asthma/COPD | ICD-9-CM | 491, 492, 496, 49300, 49301, 49302, 49310, 49311, 49312, 49320, 49321, 49322, 49381, 49382, 49390, 49391, 49392 |
|  | ICD-10-CM | J41, J42, J43, J44, J4520, J4521, J4522, J4530, J4531, J4532, J4540, J4541, J4542, J4550, J4551, J4552, J45901, J45902, J45909, J45990, J45991, J45998 |
| Hematological disorders | ICD-9-CM | 280, 281, 282, 283, 284, 285, 286, 2871, 2873, 2874, 2875 |
|  | ICD-10-CM | D46, D50, D51, D52, D53, D55, D56, D57, D58, D59, D60, D61, D62, D63, D64 |
| Dementia | ICD-9-CM | 3310, 3311, 3312, 3317, 290, 2940, 2941, 2948, 797 |
|  | ICD-10-CM | G30, G310, G311, G312, G319, F02, F03, F04, R4181 |
| Depression | ICD-9-CM | 2962, 2963, 2965, 3004, 309, 311 |
|  | ICD-10-CM | F32, F33, F341, F43 |
| Thrombocytopenia | ICD-9-CM | 286, 287 |
|  | ICD-10-CM | D68, D69 |
| AKD | ICD-9-CM | 584 |
|  | ICD-10-CM | N17 |
| Peptic ulcer diseases | ICD-9-CM | 533, V1271, |
|  | ICD-10-CM | K27, Z8711 |
| Aspirin/NSAIDs | Generic drug name | ASPIRIN, CLOPIDOGREL, CELECOXIB, DICLOFENAC, DIFLUNISAL, ETODOLAC, FENOPROFEN, FLURBUPROFEN, IBUPROFEN, INDOMETHACIN, KETOPROFEN, KETOROLAC, MEFENAMIC, MELOXICAM, NABUMETONE, NAPROXEN, OXAPROZIN, PIROXICAM, SULINDAC, TOLMETIN |
| ACEI/ARB | Generic drug name | BENAZEPRIL, CAPTOPRIL, ENALAPRIL, FOSINOPRIL, LISINOPRIL, MOEXIPRIL, PERINDOPRIL, QUINAPRIL, RAMIPRIL, TRANDOLAPRIL LOSARTAN, IRBESARTAN, OLMESARTAN, VALSARTAN, TELMISARTAN, CANDESARTAN, AZILSARTAN |
| CCB | Generic drug name | AMLODIPINE, DILTIAZEM, FELODIPINE, ISRADIPINE, LEVAMLODIPINE, NIFEDIPINE, NISOLDIPINE, VERAPAMIL |
| BB | Generic drug name | ACEBUTOLOL, ATENOLOL, BETAXOLOL, BISOPROLOL, CARVEDILOL, LABETALOL, METOPROLOL , NADOLOL, NEBIVOLOL, PINDOLOL, PROPRANOLOL, TIMOLOL |
| Antiarrhythmic drugs | Generic drug name | QUINIDINE, PROCAINAMIDE, DISOPYRAMIDE, LIDOCAINE , MEXILETINE, FLECAINIDE, PROPAFENONE, AMIODARON, EDRONEDARONE, DOFETILIDE, SOTALOL, IBUTILID, DIGOXIN |
| Diuretics | Generic drug name | HYDROCHLOROTHIAZIDE, CHLOROTHIAZIDE, CHLORTHALIDONE, EPLERENONE, FUROSEMIDE, INDAPAMIDE, SPIRONOLACTONE , TORSEMIDE, METOLAZONE |
| Statins | Generic drug name | ATORVASTATIN, FLUVASTATIN, LOVASTATIN, PITAVASTATIN, PRAVASTATIN, ROSUVASTATIN, SIMVASTATIN |
| PPIs | Generic drug name | OMEPRAZOLE, ESOMEPRAZOLE, LANSOPRAZOLE, DEXLANSOPRAZOLE, PANTOPRAZOLE, RABEPRAZOLE |
| SSRI/SNRI | Generic drug name | CITALOPRAM, ESCITALOPRAM, FLUOXETINE, FLUVOXAMINE, PAROXETINE, SERTRALINE, VILAZODONE, DULOXETINE, VENLAFAXINE, LEVOMILNACIPRAN  DESVENLAFAXINE |

**Table S2.** Characteristics of patients with new onset AFib and concomitant cancer in SEER-Medicare registry from 2012 to 2019

|  | Overall (N=39915) |
| --- | --- |
| Demographics |  |
| Index age (Mean, SD) | 77.16 (7.31) |
| Year of AFib diagnosis |  |
| 2012-2015 | 19805 (49.62) |
| 2016-2019 | 20110 (50.38) |
| Female | 18494 (46.33) |
| Race/ethnicity |  |
| Non-Hispanic White | 33971 (85.11) |
| Non-Hispanic Black | 2555 (6.40) |
| Others | 3389 (8.49) |
| Region |  |
| Midwest | 2672 (9.20) |
| Northeast | 15669 (39.26) |
| South | 7662 (19.20) |
| West | 12912 (32.35) |
| Medicaid eligible | 5739 (14.38) |
| Urbanicity |  |
| Metropolitan | 34157 (85.57) |
| Micropolitan | 3306 (8.28) |
| Unknown | 2452 (6.14) |
| Socioeconomic status (Census Tract) |  |
| Household median income (Median, IQR) | 60508.00 (44107.00- 82930.00) |
| Percentage of residents living below poverty (Median, IQR) | 9.61 (5.17-17.07) |
| Percentage of non-high school graduates (Median, IQR) | 10.01 (5.53-17.49) |
| Percentage of high school only (Median, IQR) | 27.49 (19.43-35.01) |
| Percentage of some college education (Median, IQR) | 28.54 (23.35-33.97) |
| Percentage of college education and above (Median, IQR) | 27.18 (16.44-43.18) |
| Cancer characteristics |  |
| Time from cancer diagnosis to the onset of AFib (month, Median, IQR) | 15.00 (2.00-41.00) |
| Cancer type |  |
| Breast | 10762 (26.96) |
| Lung | 17502 (42.85) |
| Prostate | 11651 (29.19) |
| Active cancer | 11601 (29.06) |
| Cancer grade |  |
| I | 4557 (11.42) |
| II | 12015 (30.10) |
| III | 12208 (30.58) |
| Others/unknown | 11135 (27.90) |
| Number of regional nodes examined |  |
| <12 | 24794 (62.12) |
| ≥12 | 2366 (5.93) |
| Unknown/missing | 12755 (31.96) |
| Tumor size |  |
| ≤2 cm | 7557 (18.93) |
| 2-5 cm | 7259 (18.19) |
| >5 cm | 3799 (9.52) |
| Unknown/missing | 21300 (53.36) |
| TMN classification |  |
| T stage |  |
| TX | 2124 (5.32) |
| T0 | 1172 (2.94) |
| T1 | 10342 (25.91) |
| T2 | 8504 (21.31) |
| T3 | 3363 (8.43) |
| T4 | 2940 (7.37) |
| Unknown/missing | 11470 (28.74) |
| N stage |  |
| NX | 1812 (4.54) |
| N0 | 18495 (46.34) |
| N1 | 2595 (6.50) |
| N2 | 4159 (10.42) |
| N3 | 1384 (3.47) |
| Unknown/missing | 11470 (28.74) |
| M stage |  |
| M0 | 22573 (56.55) |
| M1 | 5842 (14.64) |
| Unknown/missing | 11500 (28.81) |
| Summary stage |  |
| In situ | 1574 (3.94) |
| Local | 19079 (47.80) |
| Regional | 8283 (20.75) |
| Distant | 8908 (22.32) |
| Unknown/missing | 2071 (5.19) |
| Breast cancer-specific (N=10762) |  |
| ER positive | 6407 (59.53) |
| PR positive | 5516 (51.25) |
| HER2 positive status | 639 (5.94) |
| Lung cancer-specific (N=17502) |  |
| Histologic type |  |
| Adenoma | 7232 (41.32) |
| NOS | 3512 (20.07) |
| Squamous | 4867 (27.81) |
| Others | 1891 (10.80) |
| Cancer treatment |  |
| Use of potentially interacting antineoplastic agents | 7692 (19.27) |
| Radiation | 2233 (5.59) |
| Surgery | 350 (0.88) |
| Disease risk score |  |
| CHA_2_DS_2_-VASc |  |
| 1 | 3222 (8.07) |
| 2 | 6715 (16.82) |
| 3 | 9759 (24.45) |
| 4 | 10111 (25.33) |
| 5 | 6103 (15.29) |
| ≥6 | 4005 (10.03) |
| HAS-BLED |  |
| 1 | 8094 (20.28) |
| 2 | 14112 (35.36) |
| 3 | 11224 (28.12) |
| 4 | 4910 (12.30) |
| 5 | 1314 (3.29) |
| ≥6 | 261 (0.65) |
| CCI (Mean, SD) | 2.02 (2.04) |
| NCI Comorbidity score (Mean, SD) | 0.68 (0.64) |
| Individual comorbidities |  |
| Asthma/COPD | 15538 (38.93) |
| Hematological disorders | 12985 (32.53) |
| Dementia | 2671 (6.69) |
| Depression | 6602 (15.19) |
| Thrombocytopenia | 2650 (6.64) |
| Acute kidney diseases | 2768 (6.93) |
| Peptic ulcer diseases | 485 (1.22) |
| Medications |  |
| ACE inhibitors/ARBs | 12545 (31.43) |
| CCB | 7957 (19.93) |
| Beta blockers | 10694 (26.79) |
| Antiarrhythmic medications | 2122 (5.54) |
| Diuretics | 9198 (23.04) |
| Statin | 12656 (31.71) |
| PPIs | 7264 (18.20) |
| SSRIs/SNRIs | 4971 (12.45) |

AFib Atrial Fibrillation. SD Standard Deviation. IQR Interquartile Range. ER estrogen receptor. PR progesterone receptor. HER2 human epidermal growth factor receptor 2. NOS not otherwise specified. CHA_2_DS_2_-VASc: a composite score for risk of stroke. HAS-BLED a composite score for risk of bleeding. CCI Charlson Comorbidity Index. NCI National Cancer Institute Comorbidity Index. COPD Chronic obstructive pulmonary disease. ACE Angiotensin-converting enzyme. ARB Angiotensin receptor blockers. CCB Calcium Channel Blockers. PPI Pump Proton Inhibitors. SSRI Selective serotonin reuptake inhibitors. SNRI Serotonin and norepinephrine reuptake inhibitors.

**Table S3.** Distributions of time-varying unstabilized weights and truncated weights

|  | Mean | Minimum | Maximum | 95th Pctl | Std Dev | Median | 25th Pctl | 75th Pctl |
| --- | --- | --- | --- | --- | --- | --- | --- | --- |
| Stroke |  |  |  |  |  |  |  |  |
| Unstabilized weighted | 3.75 | 1.01 | 12087.07 | 11.36 | 31.02 | 1.59 | 1.31 | 2.94 |
| Unstabilized weights truncated at 99^th^ percentile | 3.21 | 1.01 | 27.28 | 11.36 | 4.20 | 1.59 | 1.31 | 2.94 |
| Major bleeding |  |  |  |  |  |  |  |  |
| Unstabilized weighted | 3.61 | 1.01 | 19456.91 | 10.86 | 36.34 | 1.60 | 1.31 | 2.84 |
| Unstabilized weights truncated at 99^th^ percentile | 3.12 | 1.01 | 26.87 | 10.86 | 4.09 | 1.60 | 3.31 | 2.84 |

Pctl percentile, Std Dev: Standard Deviation

**Table S4**. Subgroup analysis comparing 5 treatment regimens of oral anticoagulation initiation in patients with atrial fibrillation and cancer

|  | Regimen 1 | Regimen 2 | Regimen 3 | Regimen 4 | Regimen 5 |
| --- | --- | --- | --- | --- | --- |
| Subgroup analysis 1: Active cancer status | | | | | |
| *Active cancer (N =11601)* | | | | | |
| Ischemic stroke | 1.51 (1.09-2.08) | 1.53 (1.10-2.11) | 1.27 (0.90-1.79) | 0.61 (0.44-0.85) | Reference |
| Major bleeding | 0.81 (0.41-1.59) | 0.83 (0.42-1.61) | 0.59 (0.33-1.07) | 0.49 (0.41-0.60) | Reference |
| *Inactive cancer (N =28314)* | | | | | |
| Ischemic stroke | 1.26 (1.04-1.52) | 1.27 (1.05-1.54) | 1.09 (0.90-1.31) | 0.65 (0.54-0.77) | Reference |
| Major bleeding | 0.49 (0.33-0.73) | 0.56 (0.37-0.85) | 0.59 (0.39-0.87) | 0.49 (0.43-0.56) | Reference |
| Subgroup analysis 2: Cancer type | | | | | |
| *Breast cancer (N =10762)* |  |  |  |  |  |
| Ischemic stroke | 0.99 (0.78-1.25) | 0.99 (0.78-1.25) | 0.86 (0.69-1.08) | 0.50 (0.41-0.61) | Reference |
| Major bleeding | 0.38 (0.21-0.67) | 0.38 (0.22-0.68) | 0.35 (0.21-0.59) | 0.41 (0.32-0.51) | Reference |
| *Lung cancer (N=17502)* |  |  |  |  |  |
| Ischemic stroke | 2.10 (1.55-2.86) | 2.17 (1.59-2.94) | 1.94 (1.43-2.63) | 0.92 (0.64-1.33) | Reference |
| Major bleeding | 0.83 (0.43-1.64) | 0.86 (0.44-1.69) | 0.66 (0.33-1.30) | 0.47 (0.36-0.60) | Reference |
| *Prostate cancer (N=11651)* |  |  |  |  |  |
| Ischemic stroke | 0.86 (0.63-1.18) | 0.87 (0.63-1.19) | 0.61 (0.43-0.86) | 0.58 (0.47-0.70) | Reference |
| Major bleeding | 0.43 (0.24-0.77) | 0.54 (0.30-0.96) | 0.67 (0.43-1.06) | 0.59 (0.53-0.67) | Reference |
| Subgroup analysis 3: Cancer stage | | | | | |
| *In situ (N=1658)* |  |  |  |  |  |
| Ischemic stroke | 0.85 (0.46-1.58) | 0.85 (0.46-1.57) | 0.81 (0.45-1.46) | 0.36 (0.26-0.49) | Reference |
| Major bleeding | 1.01 (0.44-2.31) | 1.00 (0.44-2.30) | 1.06 (0.49-2.32) | 0.50 (0.25-1.01) | Reference |
| *Local (N=20125)* |  |  |  |  |  |
| Ischemic stroke | 0.94 (0.76-1.16) | 0.95 (0.76-1.17) | 0.75 (0.60-0.93) | 0.48 (0.41-0.56) | Reference |
| Major bleeding | 0.35 (0.22-0.55) | 0.41 (0.25-0.65) | 0.45 (0.29-0.70) | 0.43 (0.37-0.49) | Reference |
| *Regional (N=8703)* |  |  |  |  |  |
| Ischemic stroke | 1.53 (1.10-2.14) | 1.56 (1.12-2.18) | 1.28 (0.94-1.75) | 0.98 (0.70-1.39) | Reference |
| Major bleeding | 0.78 (0.35-1.76) | 0.81 (0.36-1.81) | 0.60 (0.31-1.18) | 0.55 (0.44-0.69) | Reference |
| *Distant (N=9411)* |  |  |  |  |  |
| Ischemic stroke | 2.09 (1.33-3.28) | 2.14 (1.37-3.35) | 2.18 (1.37-3.45) | 0.95 (0.54-1.67) | Reference |
| Major bleeding | 1.17 (0.48-2.90) | 1.20 (0.49-2.98) | 0.81 (0.31-2.17) | 0.54 (0.40-0.73) | Reference |
| Subgroup analysis 4: Tumor grade | | | | | |
| *Grade I (N=4557)* |  |  |  |  |  |
| Ischemic stroke | 0.92 (0.60-1.41) | 0.93 (0.61-1.42) | 0.67 (0.45-1.02) | 0.52 (0.32-0.83) | Reference |
| Major bleeding | 0.59 (0.25-1.40) | 0.60 (0.26-1.42) | 0.40 (0.21-0.79) | 0.29 (0.20-0.41) | Reference |
| *Grade II (N=12015)* |  |  |  |  |  |
| Ischemic stroke | 1.07 (0.82-1.39) | 1.10 (0.84-1.43) | 0.98 (0.75-1.28) | 0.60 (0.47-0.76) | Reference |
| Major bleeding | 0.27 (0.14-0.50) | 0.28 (0.15-0.53) | 0.33 (0.21-0.52) | 0.45 (0.38-0.54) | Reference |
| *Grade III (N=12208)* |  |  |  |  |  |
| Ischemic stroke | 1.14 (0.81-1.59) | 1.13 (0.81-1.59) | 1.02 (0.72-1.43) | 0.66 (0.48-0.91) | Reference |
| Major bleeding | 0.53 (0.29-0.96) | 0.67 (0.34-1.29) | 0.75 (0.41-1.38) | 0.58 (0.48-0.68) | Reference |

********Effect measure was average hazard ratio (HR) and 95% Confidence Interval (95% CI) over 12-month follow-up, adjusted for baseline and time-varying covariates*

*Regimen 1: Initiate oral anticoagulants when CHA_2_DS_2_-VASc score ≥1
Regimen 2: Initiate oral anticoagulants when CHA_2_DS_2_-VASc score ≥2
Regimen 3: Initiate oral anticoagulants when CHA_2_DS_2_-VASc score ≥4
Regimen 4: Initiate oral anticoagulants when CHA_2_DS_2_-VASc score ≥6
Regimen 5: Never initiate oral anticoagulants*

**Table S5**. Sensitivity analysis comparing 5 treatment regimens of oral anticoagulation initiation in patients with atrial fibrillation and cancer

|  | Regime 1 | Regime 2 | Regime 3 | Regime 4 | Regime 5 |
| --- | --- | --- | --- | --- | --- |
| Sensitivity analysis 1 (N=39915) | | | | | |
| Ischemic stroke | 1.25 (1.07-1.46) | 1.27 (1.09-1.49) | 1.18 (1.00-1.39) | 0.84 (0.70-1.01) | Reference |
| Major bleeding | 0.76 (0.59-0.98) | 0.79 (0.61-1.02) | 0.71 (0.56-0.91) | 0.47 (0.41-0.54) | Reference |
| Sensitivity analysis 2 (N=30504) | | | | | |
| Ischemic stroke | 1.07 (0.90-1.26) | 1.08 (0.91-1.28) | 0.88 (0.74-1.04) | 0.58 (0.50-0.67) | Reference |
| Major bleeding | 0.48 (0.34-0.68) | 0.53 (0.37-0.77) | 0.55 (0.39-0.77) | 0.47 (0.42-0.52) | Reference |
| Sensitivity analysis 3 (N=37265) | | | | | |
| Ischemic stroke | 1.32 (1.11-1.56) | 1.34 (1.13-1.58) | 1.10 (1.93-1.32) | 0.63 (0.54-0.75) | Reference |
| Major bleeding | 0.58 (0.40-0.83) | 0.63 (0.44-0.92) | 0.60 (0.42-0.85) | 0.49 (0.44-0.56) | Reference |
| Sensitivity analysis 4 (N=39915) | | | | | |
| Stroke | 1.27 (1.09-1.47) | 1.27 (1.10-1.48) | 0.99 (0.86-1.15) | 0.56 (0.50-0.63) | Reference |
| Major bleeding | 0.52 (0.39-0.72) | 0.56 (0.41-0.76) | 0.53 (0.41-0.68) | 0.51 (0.46-0.57) | Reference |

********Effect measure was average hazard ratio (HR) and 95% Confidence Interval (95% CI) over 12-month follow-up, adjusted for baseline and time-varying covariates*

*Regime 1: Initiate oral anticoagulants when CHA_2_DS_2_-VASc score ≥1
Regime 2: Initiate oral anticoagulants when CHA_2_DS_2_-VASc score ≥2
Regime 3: Initiate oral anticoagulants when CHA_2_DS_2_-VASc score ≥4
Regime 4: Initiate oral anticoagulants when CHA_2_DS_2_-VASc score ≥6
Regime 5: Never initiate oral anticoagulants*

*Sensitivity analysis 1. Extending maximum follow-up to 36 months
Sensitivity analysis 2. Excluding patients with metastatic cancer at baseline
Sensitivity analysis 3. Excluding patients with thrombocytopenia at baseline
Sensitivity analysis 4. Truncating stabilized weights at 95^th^ percentile*

**
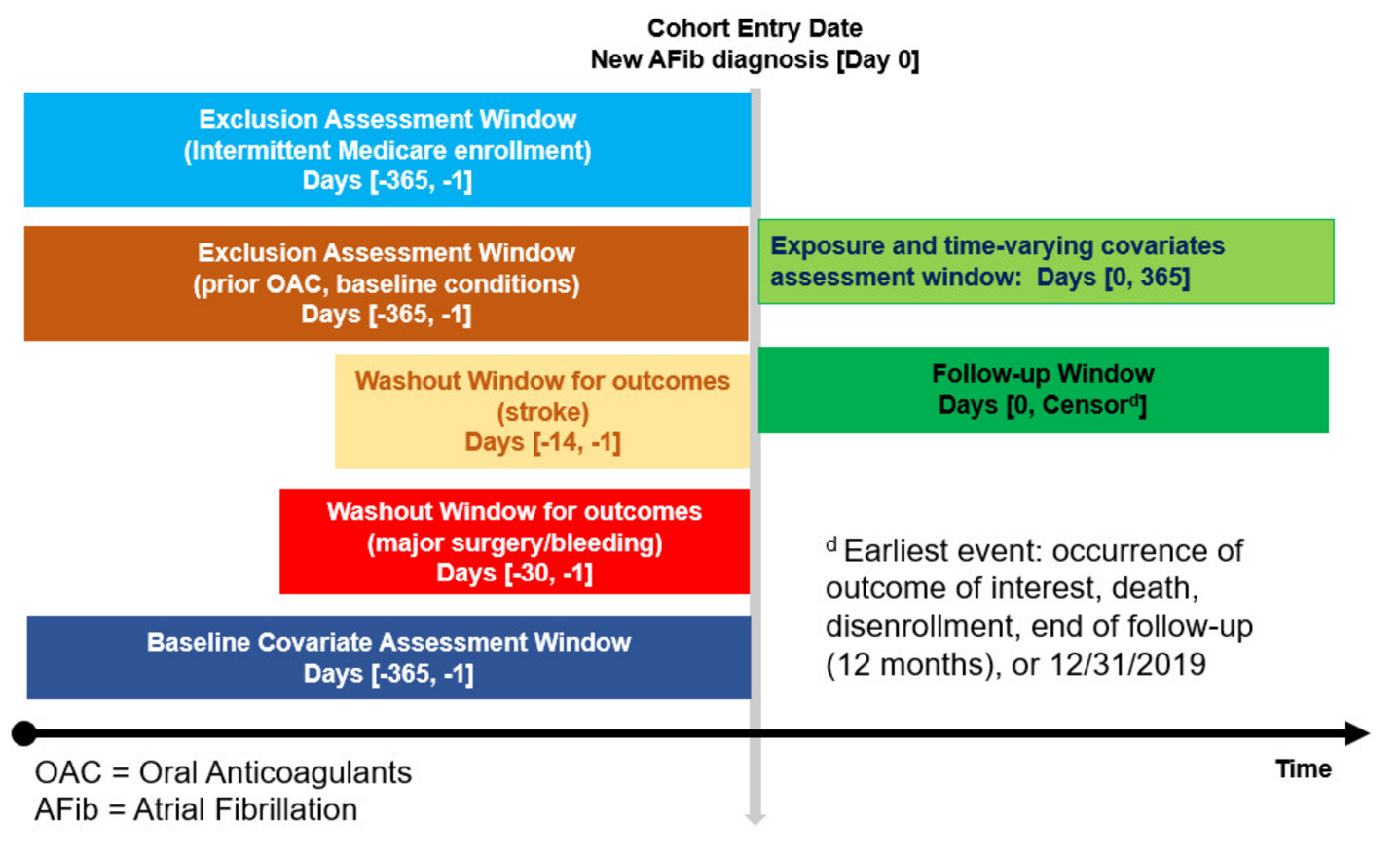
**

**Figure S1**. Visualization of study timeline


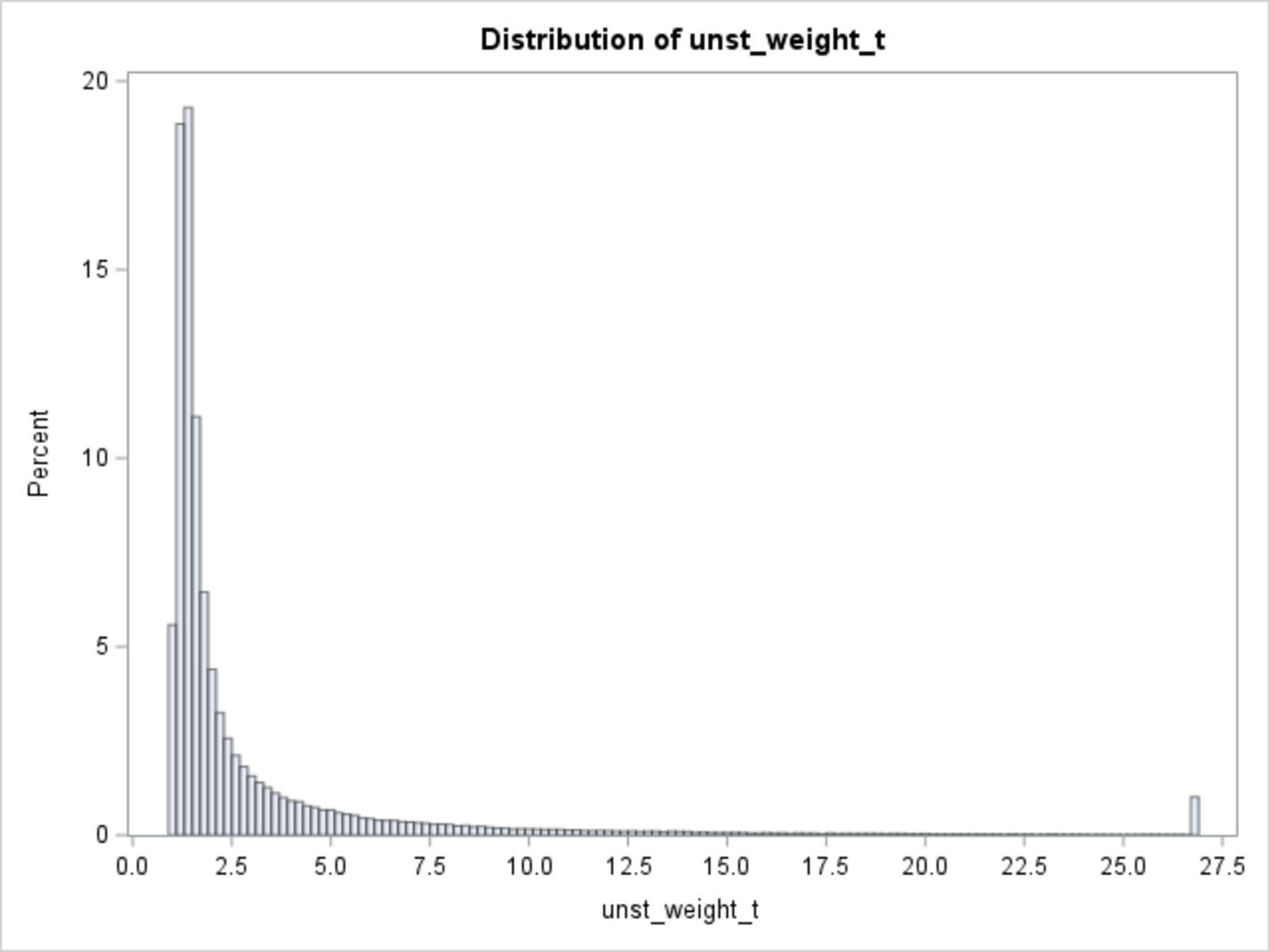


**Figure S2**. Distribution of unstabilized weights truncated at 99^th^ percentile in the analysis of ischemic stroke

**
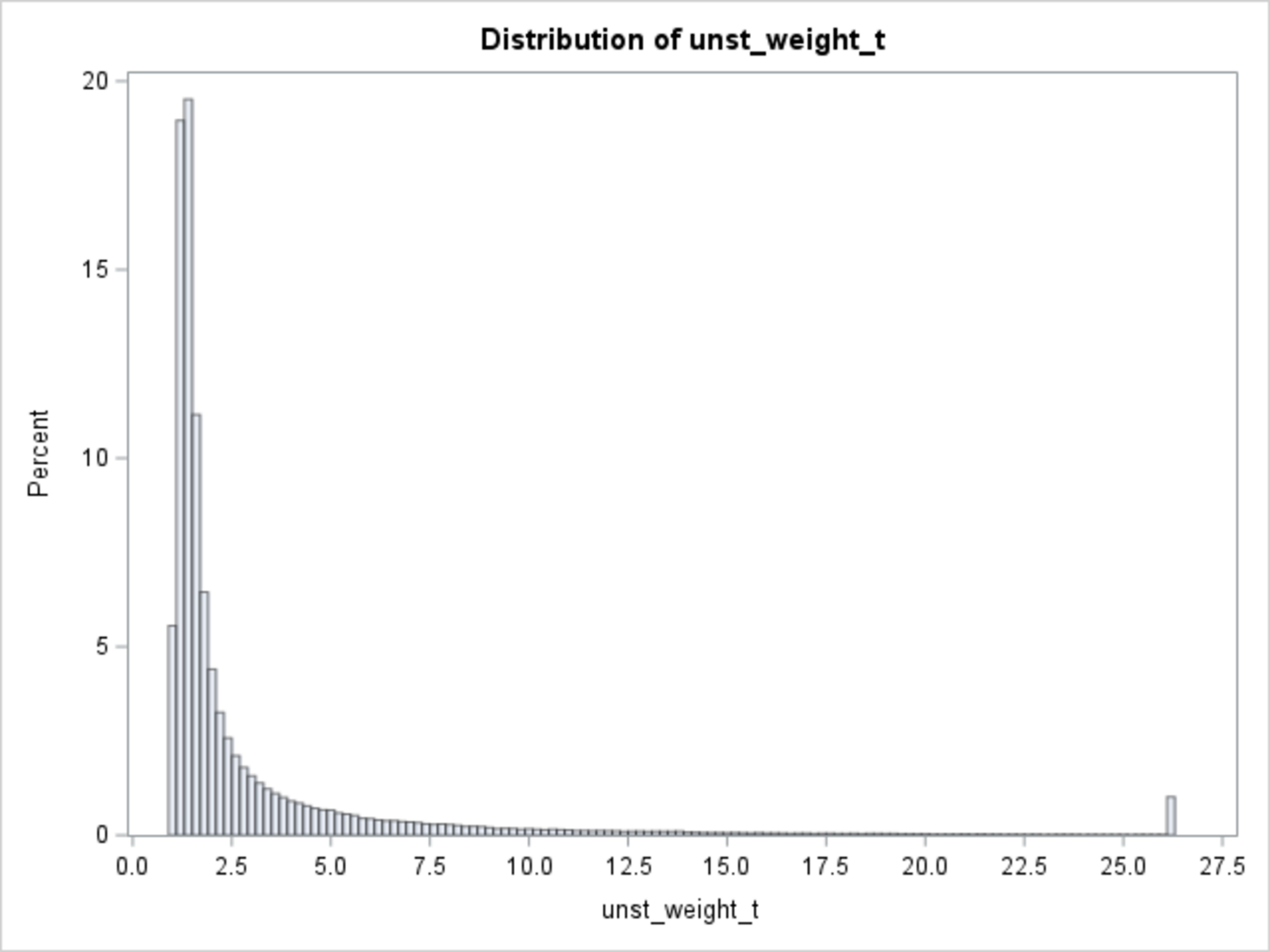
**

**Figure S3**. Distribution of unstabilized weights truncated at 99^th^ percentile in the analysis of major bleeding

**Technical appendix^1-6^**

Step 1 – cloning: First, we organized each persons’ data in discrete-time (person-month) format (month 1 to 12). We then duplicated each individual’s data 5 times and assigned them to 5 different treatment strategies. The stacked data set is 5 times as large as the original sample. At this point, there was no difference in baseline covariates between 5 treatment groups as replicates are identical and no confounder exists. At baseline, some of the replicates already passed the cut-off values of CHA_2_DS_2_-VASc score to start OACs without starting OAC were removed from the dataset and vice versa. These patients were removed from the analysis. For example, a replicate of a patient with baseline CHA_2_D_2_VASc score of 4 with treatment strategy of "initiate OACs when CHA_2_DS_2_-VASc score ≥1" without OAC prescription in the first month were deleted.

Step 2 – Censoring: In this step, replicates whose data no longer consistent with their assigned strategy during follow-up were censored. We checked whether replicates in each month adhered to their assigned strategy and censored them if they did not adhere. In particular, suppose that individuals with CHA_2_DS_2_VASc score of 2 at baseline were assigned to treatment strategies “initiate OACs when CHA_2_DS_2_-VASc score ≥4”. These individuals were censored if they received the treatment when their CHA_2_D_2_VASc score remained or rose to 3 at a specific month during follow-up. However, these patients were not censored if they remain untreated at CHA_2_D_2_VASc score is 2 or 3 or start OACs when their CHA_2_D_2_VASc score increases to 4.

Step 3 – Weighting: Because artificial censoring is informative and this informative censoring introduces selection bias, we weighed those who were artificially censored by IP weighting to reduce selection bias. That is, uncensored replicates have an equal weight as the inverse probability of remaining uncensored, given their own covariate history. The purpose is to upweight the uncensored replicates so that in the pseudo-population, censoring does not depend on baseline fixed-time and time-varying covariates and censoring is no longer informative. To calculate the weights due to protocol violation, we need to calculate the probability of being censored, which is a product of the probability of starting OACs and by the probability of not being censored due to loss to follow-up.

Specifically, the censoring weight of each replicate at time t is proportional to the probability of being uncensored through time t conditioning on not having the outcome before time t ($Y_{t-1}=0)$, fixed-time covariates ($L_{0}$) and time-varying covariates ($\bar{L_{t}}$), treatment history ($\bar{A}_{t-1}$), where $A_{t}$= 1 indicates that an individual has received OACs and $A_{t}$= 1 denotes an untreated status by week t.^2^ Since the censoring status of individuals depends on OAC initiation, we estimated the probability of remaining uncensored by the probability of initiating OACs for each individual at each month.^2,3,6,7^ In the original dataset, we fitted a logistic regression as follow:

$logit(\Pr\left[ A_{k}=1 | A_{k-1}=0, \bar{L}_{k}, \bar{Y}_{t-1}=0, L_{0}, L_{k} \right])$ = $\theta_{0}$ + $\theta_{1}^{T}L_{0}$ +$\theta_{2}^{T}L_{k}$

where the overline (i.e., $\bar{L}_{k}$) represents the history of covariate from the start of follow-up, the superscript T indicates a transpose of a vector of coefficients, $\theta_{0}$ is a time-specific intercept (estimated via linear and quadratic terms for t), $L_{0}$ is the vector of baseline covariates, and $L_{k}$ is the vector of time-varying covariates through time $k$, a is an indicator each treatment regimen, and $i$ indicates the number of emulated trials. The unstabilized time-varying inverse-probability weight is computed by:

$W_{i,t}^{a}$ = $\prod_{k=0}^{t} \frac{1}{f\left( A_{k} \right|\bar{A}_{k-1},\bar{Y}_{k-1}=0,\bar{C}_{k-1}=0, \bar{L}_{k}, L_{0})}$

We also addressed selection bias due to lost to follow-up and death. Separate logistic regression models for each treatment strategy were constructed to estimate the probability of being lost to follow-up or death, given baseline and time-varying covariates. In the unexpanded dataset, the unstabilized natural censoring weights for loss-to-follow-up (W^LFU^) for each individual at time t were calculated by

$W_{i,t}^{n, LFU}$ = $\prod_{k=0}^{t} \frac{1}{f\left( C_{k} \right|\bar{C}_{k-1}=0,\bar{Y}_{k-1}=0,\bar{A}_{k-1}=0, \bar{L}_{k}, L_{0} )}$

where $C_{k}$ is an indicator of natural censoring.^6,7^ The final weights are the products of the probability of starting OACs and by the probability of not being censored due to loss to follow-up for each person-month. We truncated weights at 99^th^ percentile to avoid extreme weights.

$W_{total}$ = $W_{i,t}^{a}$ x ${SW}_{i,t}^{n, LFU}$

Under the assumption that (i) our measured baseline and time-varying confounders are sufficient to ensure exchangeability, (ii) positivity – that is the probability of each individual is greater than 0 within levels of covariates dynamic treatment effect, and (iii) weighted models are correctly specified, the treatment effect of each treatment regimen can be estimated using a marginal structural model (MSM).

$logit(\Pr\left[ Y_{t+1}^{a}=1 | L_{0}, Y_{t}^{a}=0 \right])$ = $\gamma_{0}$ + $\gamma_{1}^{T}L_{0}$ +$\gamma_{2}a$

where $Y_{t}^{a}$is the counterfactual outcome at month t under the strategy a=A.

To estimate the treatment effect, a weighted pooled logistic regression model were used to estimate the per-protocol effect of 4 "active" treatment strategies compared to the reference strategy. The regressors include f(t) – a function of time and time squared (time and time squared ), treatment strategy, interactions between time and treatment, and all baseline covariates, weighted for censoring ($W_{total}$)

$logit(\Pr\left[ Y_{k+1}=1 | A, L_{0}, \bar{Y}_{k}=0 \right])$ = $\beta_{0}$ + $\beta_{1}^{T}L_{0}$ +$\beta_{2}$*A* + f(t)

The pooled logistic regression is equivalent to discrete-time hazard model, with the assumption that the risk of developing a specific outcome of interest within levels of covariates is rare in each time interval (risk <0.1).^8,9^ The odds ratio of developing the event is equivalent to the PP HR, estimated by exponentiated coefficient of treatment indicator ($e^{\beta_{2}})$.^8,9^

To estimate the weighted survival curves and standardize the curves to baseline distribution of covariates, we fitted a similar model with an interaction term between treatment regimen A and f(t) – a function of time and time squared to allow for non-proportional hazards. The model was then used to predict outcomes of interest under each treatment strategy $A=a$. The details for creating standardized survival curves can be found in published studies.^10^

**References**

1. Faries D, Zhang X, Kadziola Z. *Real World Health Care Data Analysis: Causal Methods and Implementation Using SAS®.* SAS Institute; 2020.

2. Cain LE, Robins JM, Lanoy E, Logan R, Costagliola D, Hernán MA. When to start treatment? A systematic approach to the comparison of dynamic regimes using observational data. *Int J Biostat.* 2010;6(2):Article 18.

3. Cain LE, Saag MS, Petersen M, et al. Using observational data to emulate a randomized trial of dynamic treatment-switching strategies: an application to antiretroviral therapy. *International Journal of Epidemiology.* 2016;45(6):2038-2049.

4. Hernán MA. How to estimate the effect of treatment duration on survival outcomes using observational data. *BMJ (Clinical research ed).* 2018;360:k182-k182.

5. Hernán MA, Robins JM. *Causal Inference: What If.* Boca Raton: Chapman & Hall/CRC; 2020.

6. Lyu H, Yoshida K, Zhao SS, et al. Delayed Denosumab Injections and Fracture Risk Among Patients With Osteoporosis : A Population-Based Cohort Study. *Ann Intern Med.* 2020;173(7):516-526.

7. Douglas Faries, Xiang Zhang, Zbigniew Kadziola, et al. Chapter 12: A Target Trial Approach with Dynamic Treatment Regimes and Replicates Analyses. In: *Real World Health Care Data Analysis: Causal Methods and Implementation Using SAS®: Causal Methods and Implementation Using SAS®.* Cary, NC: SAS Institute; 2020.

8. Thompson WA, Jr. On the treatment of grouped observations in life studies. *Biometrics.* 1977;33(3):463-470.

9. D'Agostino RB, Lee ML, Belanger AJ, Cupples LA, Anderson K, Kannel WB. Relation of pooled logistic regression to time dependent Cox regression analysis: the Framingham Heart Study. *Stat Med.* 1990;9(12):1501-1515.

10. Murray EJ, Caniglia EC, Petito LC. Causal survival analysis: A guide to estimating intention-to-treat and per-protocol effects from randomized clinical trials with non-adherence. *Research Methods in Medicine & Health Sciences.* 2020;2(1):39-49.
